# Identification and validation of circadian rhythm and astrocyte-associated diagnostic and therapeutic model for cirrhosis encephalopathy patients via integrative bioinformatic pipelines and *in vitro* validation

**DOI:** 10.3389/fnins.2026.1897110

**Published:** 2026-07-30

**Authors:** Nianshan Ji, Yiyao Cheng, Yuheng Peng, Zhiguang Sun

**Affiliations:** The First Clinical Medical College, Nanjing University of Chinese Medicine, Nanjing, China

**Keywords:** artificial intelligence, astrocytes, circadian rhythm, cirrhosis encephalopathy, *in vitro* validation, multi-omics

## Abstract

**Background:**

Cirrhosis encephalopathy (CE) is a severe neuropsychiatric complication of liver cirrhosis, characterized by cognitive decline. While circadian rhythm (CR) disruption and astrocyte dysfunction are independently implicated, their integrated role in CE pathogenesis remains elusive.

**Methods:**

Limma and WGCNA analysis were performed for identification of CR and astrocyte (CA)-associated DEGs in CE patient bulk data (GSE41919 and GSE53808). Next, in 2 dependent CE patient bulk data (GSE184220 and GSE149741), we pinpointed CA-associated hub gene and nominated its corresponding diagnostic potential for CE patients via random forest (RF) machine learning algorithm. Next, molecular and immune patterns of hub gene in CE were examined in GSE184220 via single-gene GSEA and CIBERSORT analysis. Single-cell RNA-seq (GSE163577) from cognitive impairment patients was used to validate the cellular specificity of the hub gene and its functional implications in astrocyte via cutting-edge analytical framework, such as monocle2 and scTenifoldKnk analysis. Artificial intelligence (AI)-driven framework (DrugReflector) coupled with molecular docking identified a therapeutic compound in GSE41919 for the treatment of CE. Finally, an *in vitro* CE model using SVGp12 cells was used for the examination of hub gene expression.

**Results:**

IL8 can be considered as up-regulated CA-associated pathogenic factor involved in the pathogenesis of CE, which was predominantly active in astrocytes and related to neuroinflammation and CR regulation. AI-based drug screening nominated BRD-K11973162 as a potential therapeutic compound.

**Conclusion:**

This study discovered CA-related molecular patterns CE. IL18 emerges as a central pathogenic factor within astrocytes, providing a novel framework for risk stratification and targeted therapy for this debilitating condition.

## 1 Introduction

Liver cirrhosis (LC), the end-stage of chronic liver disease, is a major global health burden (Barnett, 2018). Hepatic encephalopathy (CE) is a severe neuropsychiatric complication of cirrhosis, characterized by a spectrum of neurological and psychiatric abnormalities ranging from subclinical cognitive impairment to coma (Rudler et al., 2021). The pathogenesis of CE is multifactorial, primarily driven by the accumulation of gut-derived neurotoxins, most notably ammonia, which cannot be cleared by the failing live (Wijdicks, 2016). These toxins enter the brain and disrupt astrocyte function, leading to cerebral edema, altered neurotransmission, and systemic inflammation (Ohikere and Wong, 2024). Epidemiologically, the prevalence of overt CE in cirrhotic patients is substantial, and its occurrence is a strong predictor of poor prognosis and increased mortality (Krishnarao and Gordon, 2020). Despite current treatments like lactulose and rifaximin, the residual risk of recurrent episodes remains high, underscoring the urgent need to understand the specific molecular pathways that trigger this neurological decline (Badal and Bajaj, 2023).

Recent evidence has highlighted the critical role of circadian rhythm (CR) disruption in the pathophysiology of chronic liver disease and its neurological complications (Ishay et al., 2021). CR genes regulate a wide array of physiological processes, including the sleep-wake cycle, metabolism, and immune function (Velissaris et al., 2008). Dysregulation of the core clock machinery is known to exacerbate neuroinflammation and astrocyte dysfunction (Cheon and Song, 2021). Concurrently, astrocytes, the most abundant glial cells in the brain, play a crucial role in maintaining homeostasis at the blood-brain barrier (BBB) and supporting neuronal function (Popek et al., 2025). In the context of CE, ammonia-induced astrocyte swelling and metabolic impairment are key pathological events (Sen et al., 2025). While both CR disruption and astrocyte dysfunction are independently linked to CE, a molecular axis that integrates these two pathways has not been established.

To address this gap, we identified that a CA-associated molecular signature regulated drives the pathogenesis of CE. Here, we conducted a comprehensive, AI-augmented multi-omics analysis of brain and blood transcriptomes. By integrating bulk transcriptomics, single-cell resolution mapping, and *in vitro* validation, we aimed to identify a core CA-related gene signature, characterize its cellular specificity within astrocytes, and nominate a potential therapeutic target for halting disease progression in CE patients.

## 2 Material and methods

### 2.1 Source of data

Transcriptomic datasets were retrieved from the Gene Expression Omnibus (GEO) database using the GEOquery package in R (Tu et al., 2023). The internal discovery set for brain tissue was GSE41919, comprising 3 LC without CE and 8 LC with CE samples was used for Limma analysis (Hsu et al., 2021). GSE53808 (15 LC without CE and 8 LC with CE brain samples) was used for xCell and WGCNA analysis (Sutherland et al., 2014). The training set for the diagnostic model was GSE184220 (6 LC without CE and 6 LC with CE blood samples), with GSE149741 (5 LC without CE and 6 LC with CE blood samples) as the validation set (Li et al., 2024; Rubio et al., 2021). A curated list of CR-related genes was compiled from the GeneCards database (relevance score > 1).

### 2.2 Limma analysis

Differential expression analysis between LC without CE and LC with CE groups in GSE41919 was performed using the limma package in R (Ritchie et al., 2015). Genes meeting a threshold of an absolute log2 fold change (| log2FC|) > 1 and a *p*-value < 0.05 were considered significant DEGs (Ritchie et al., 2015). These DEGs were intersected with the CR gene list to identify CR-associated DEGs. GO and KEGG enrichment analyses were performed using the clusterProfiler package in R, referencing the KEGG and GO gene sets from the MSIGDB database (Yu et al., 2012). Expression patterns of CR-associated DEGs were visualized with a heatmap using the complexheatmap package in R (Gu, 2022).

### 2.3 xCell and WGCNA analysis

The xCell algorithm in R was applied to GSE53808 to estimate the enrichment score of astrocytes in each sample (He et al., 2025). To identify gene co-expression networks associated with astrocyte abundance, WGCNA was performed on GSE53808 using the WGCNA package in R (Langfelder and Horvath, 2008). A soft-thresholding power (β) was selected to achieve a scale-free topology fit index (R^2^) > 0.85 (Langfelder and Horvath, 2008). The module showing the strongest Pearson correlation with the astrocyte score was selected as the astrocyte-correlated module (Langfelder and Horvath, 2008). These module genes were then intersected with the CR-associated DEGs to define the CA-associated shared DEGs. Friend analysis of these genes was performed using the GOSemSim package in R, followed by GO and KEGG enrichment in R HEto identify their primary biological functions referencing the KEGG and GO gene sets from the MSIGDB database (Yu, 2020; Zhou et al., 2026).

### 2.4 Random forest machine learning for diagnostic model construction

A diagnostic model for CE was constructed using the caret package in R, specifically implementing the random forest (RF) algorithm (Jin et al., 2023). Specifically, for the RF threshold setting, Briefly, the following RF parameters were as: ntree = 1000, mtry = 10 (optimized by repeated cross-validation), and nodesize = 5. The normalized expression values of the CA-associated DEGs were used as input features (Jin et al., 2023). The model was trained on GSE184220 using 10-fold cross-validation (Wallace et al., 2023). Model performance was evaluated by the mean area under the receiver operating characteristic curve (AUC-ROC) (Wallace et al., 2023). ROC and PR curves were performed via the pROC package in R in both GSE149741 and GSE184220 (Robin et al., 2011). Single-gene GSEA was performed for IL18 in GSE184220 using the clusterProfiler package in R, referencing the KEGG and GO gene sets from the MSIGDB database (Yu et al., 2012). Immune cell infiltration correlation of hub gene with immune cells was assessed using the CIBERSORT algorithm in R in GSE184220 (Chen et al., 2018).

### 2.5 Single-cell analysis

The scRNA-seq data from cognitive impairment patients (GSE163577, 9 patient brain samples) were processed using the Seurat package in R (He et al., 2022; Tan et al., 2023). After quality control (200–6000 features, < 15% mitochondrial content), cells were normalized, scaled, and clustered (Tan et al., 2023). Cell types were annotated using the SingleR package in R with the Human Primary Cell Atlas reference (Cao et al., 2023). Cell-cell communication was inferred using the CellChat package in R (Fang et al., 2022). Metabolic heterogeneity was assessed using the scMetabolism package in R (Argüello et al., 2020). The expression of hub gene across cell types was visualized on a feature plot. A virtual knockout of hub gene specifically within the specific cell cluster was simulated using the scTenifoldKnk package in R, followed by GO and KEGG enrichment analysis referencing the KEGG and GO gene sets from the MSIGDB database and GENEMINIA analysis of the perturbed genes (Osorio et al., 2022; Zhao et al., 2021). Pseudotime trajectory analysis of astrocytes was performed using the Monocle2 package in R (Liu et al., 2025).

### 2.6 Drug screening and molecular docking

The artificial intelligence (AI)-driven DrugReflector platform was applied to the CA-associated gene expression signature from GSE184220 to screen the CMAP database for compounds that could reverse the disease state (Chen et al., 2026). The top-ranked compound was selected (Chen et al., 2026). Molecular docking simulations were performed using AutoDock Vina software (Chen et al., 2026). The 3D structure of the hub protein was obtained from the PDB database, and the compound structure from PubChem database (Chen et al., 2026). The conformation with the lowest binding energy was visualized using PyMOL software (Chen et al., 2026).

### 2.7 Cell lines and culture conditions

The SVGp12 cell line used in this study was obtained from SUNNCELL company (China). After thawing and recovery, SVGp12 cells were cultured in DMEM/F12 medium containing 10% FBS and maintained at 37 °C in a humidified incubator with 5% CO2. Once cell density reached 75%–85% confluence, the cells were sub-cultured, passaged, and cryopreserved as needed. A fatty acid stock solution was prepared by combining sodium oleate and sodium palmitate at a 2:1 molar ratio, which was then diluted to a final concentration of 1.2 mmol/L to form the fatty acid induction medium. SVGp12 cells were assigned to either a control group or a CE model group. Cells in the control group received standard complete medium, whereas those in the model group were exposed to the 1.2 mmol/L fatty acid induction medium. After 24 h of treatment, relevant cellular parameters were assessed for both groups.

### 2.8 Q-RT-PCR

Total RNA was extracted using TRIzol (Takara, Japan), and cDNA was synthesized with the PrimeScript RT reagent Kit (Vazyme, China). q-RT-PCR was performed using TB Green Premix Ex Taq II (Vazyme, China) on a QuantStudio 5 system (Applied Biosystems, USA). Relative target gene expression was calculated by the 2^–^ΔΔCt method. Primer sequences (5′→3′) used were:

IL18:

F:5′-GCTTCCTCTCGCAACAAACT-3′
R: 5′-GTTCCTCCATACCTTGACAGTTG-3′

GAPDH:

F: 5′-GGAGCGAGATCCCTCCAAAAT-3′
R: 5′-GGCTGTTGTCATACTTCTCATGG-3′.

### 2.9 Statistical analysis

All statistical analyses were conducted using R (version 4.2.2) or GraphPad Prism (version 9.0). For two-group comparisons, an unpaired two-tailed Student’s *t*-test or Mann-Whitney U test was used as appropriate. A *p*-value < 0.05 was considered statistically significant.

## 3 Results

### 3.1 Identification of CR-associated DEGs for CE patients

**FIGURE 1:**
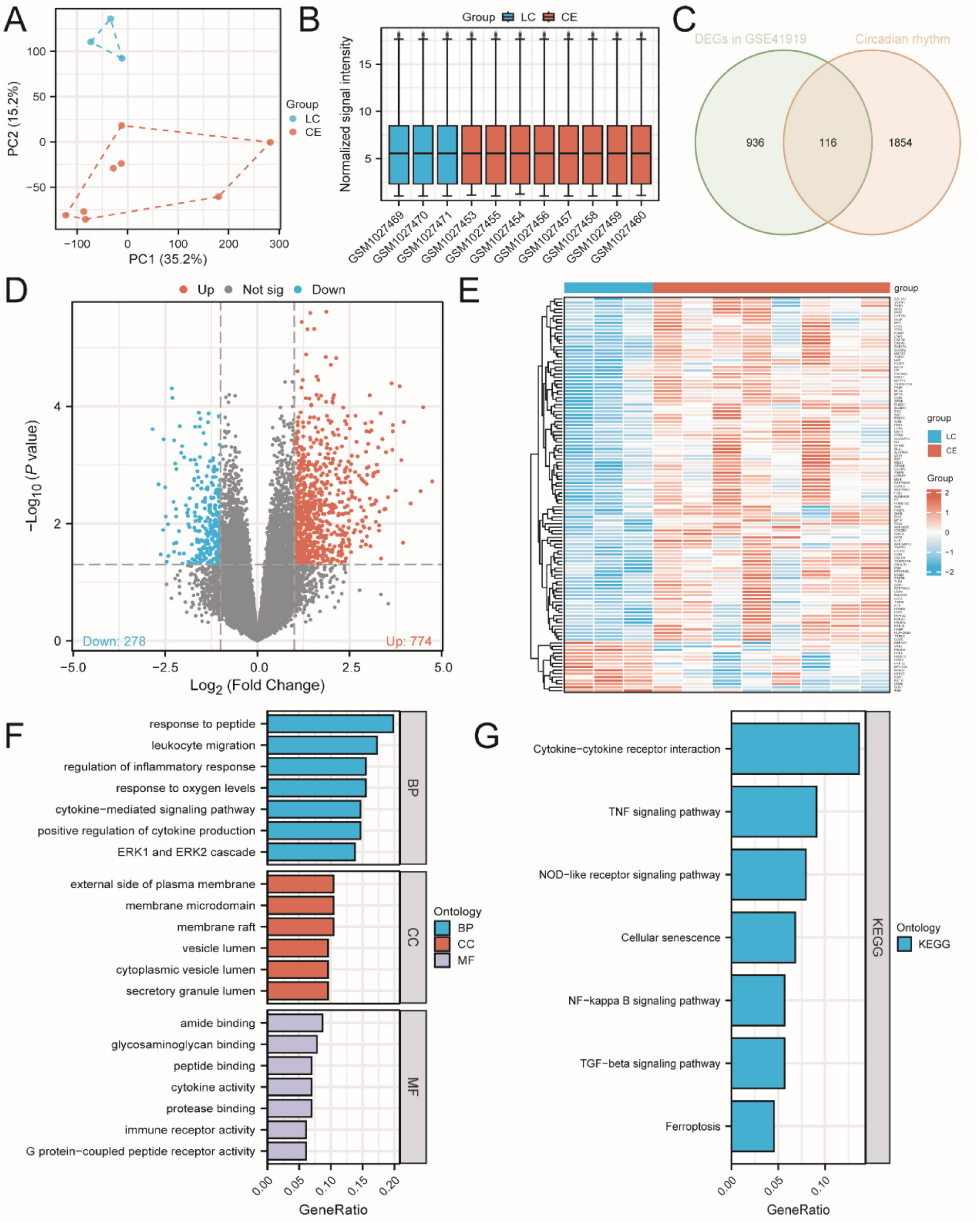
Identification of CR-associated DEGs in CE. **(A)** PCA plot in GSE41919. **(B)** Box plot in GSE41919. **(C)** Venn diagram showing the intersection of DEGs from GSE41919 with the CR gene list, yielding 116 CR-associated DEGs. **(D)** Volcano plot in GSE41919. **(E)** Heatmap of the 116 CR-associated DEGs, showing distinct expression patterns between LC and CE groups. **(F,G)** Bar plots of enriched GO and KEGG terms for the CR-associated DEGs.

To explore the molecular basis of CE, we first pre-processed the brain transcriptome of GSE41919 (Figures 1A,B). Differential expression analysis identified DEGs between CE and LC samples (Figure 1D). The intersection of these DEGs with the CR gene list yielded 116 CR-associated DEGs (Figure 1C). A heatmap of these 116 genes revealed a distinct expression pattern that effectively segregated the CE and LC groups (Figure 1E). GO and KEGG enrichment analysis of these CR-associated DEGs highlighted their involvement in pathways related to neuroinflammation and epigenetic modification (Figures 1F,G).

### 3.2 Identification of CA-associated DEGs for CE patients

**FIGURE 2:**
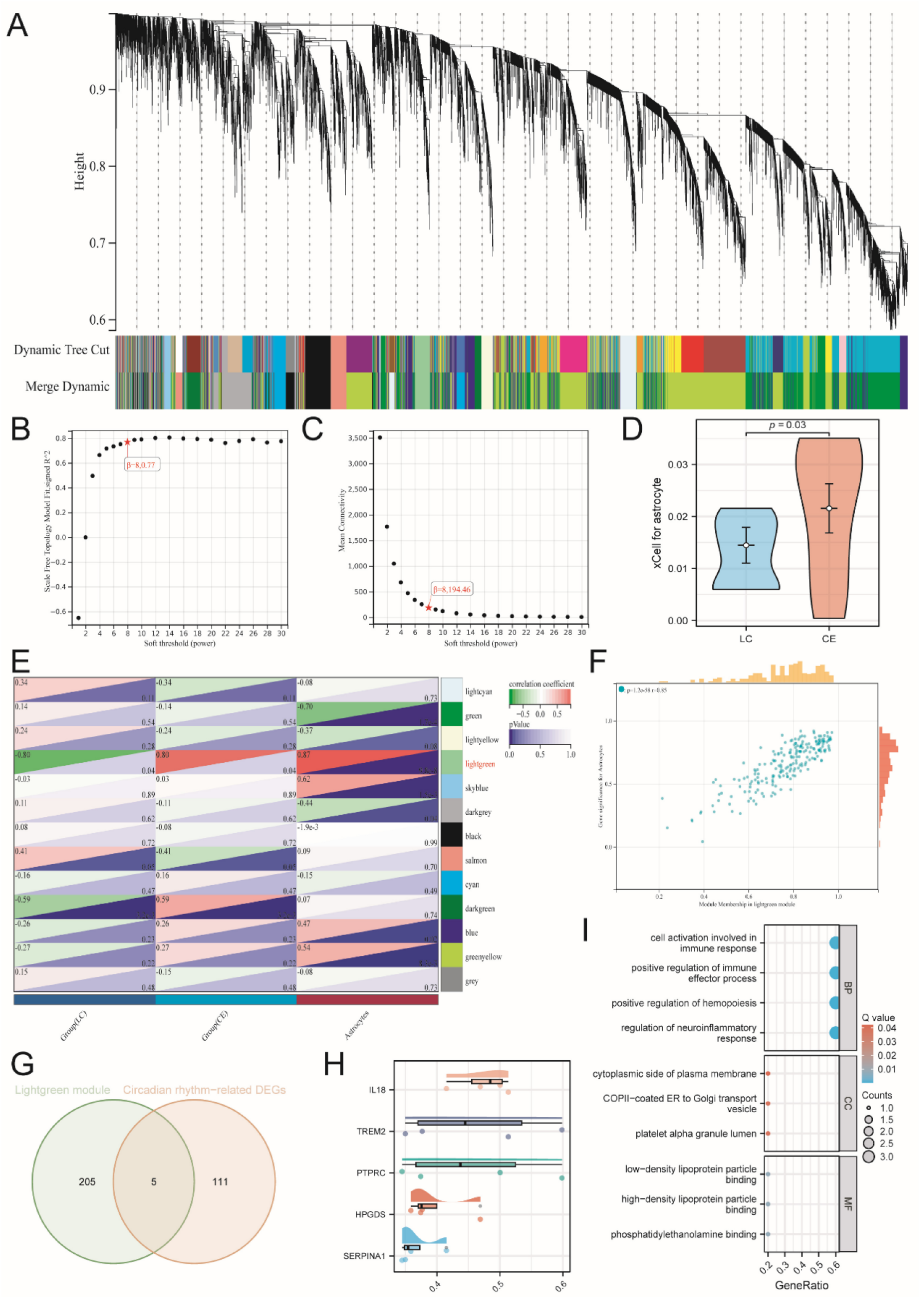
Identification of the CA signature in CE. **(A–C** and **E,F)** Module-trait relationships heatmap from WGCNA, highlighting the lightgreen module as most correlated with astrocyte score and disease status. **(D)** Box plot from xCell analysis showing a higher astrocyte score in CE patients. **(G)** Venn diagram showing the intersection of 116 CR-associated DEGs and genes from the lightgreen module, yielding 5 CA-associated DEGs. **(H)** Friend analysis of the 5 CA-associated DEGs. **(I)** GO and KEGG enrichment of the 5 CA-associated DEGs.

xCell analysis of GSE53808 revealed a significantly higher enrichment score for astrocytes in CE patients compared to LC controls (Figure 2D). To identify genes correlated with this astrocyte abundance, we performed WGCNA (Figure 2A). A soft threshold of β = 8,0.77 and 8,194.46 were selected to achieve a scale-free topology (Figures 2B,C). The lightgreen module showed the strongest positive correlation with both the astrocyte score and CE disease status (Figures 2E,F). Intersecting the 116 CR-associated DEGs with the genes from the lightgreen module yielded 5 core CA-associated shared DEGs, including IL18, TREM2, PTPRC, HPGDS, and SERPINA1 (Figure 2G). Friend analysis showed a strong functional relationship among these genes (Figure 2H), and enrichment analysis confirmed their primary association with neuroinflammation (Figure 2I).

### 3.3 Identification of CA-associated diagnostic model for CE patients

**FIGURE 3:**
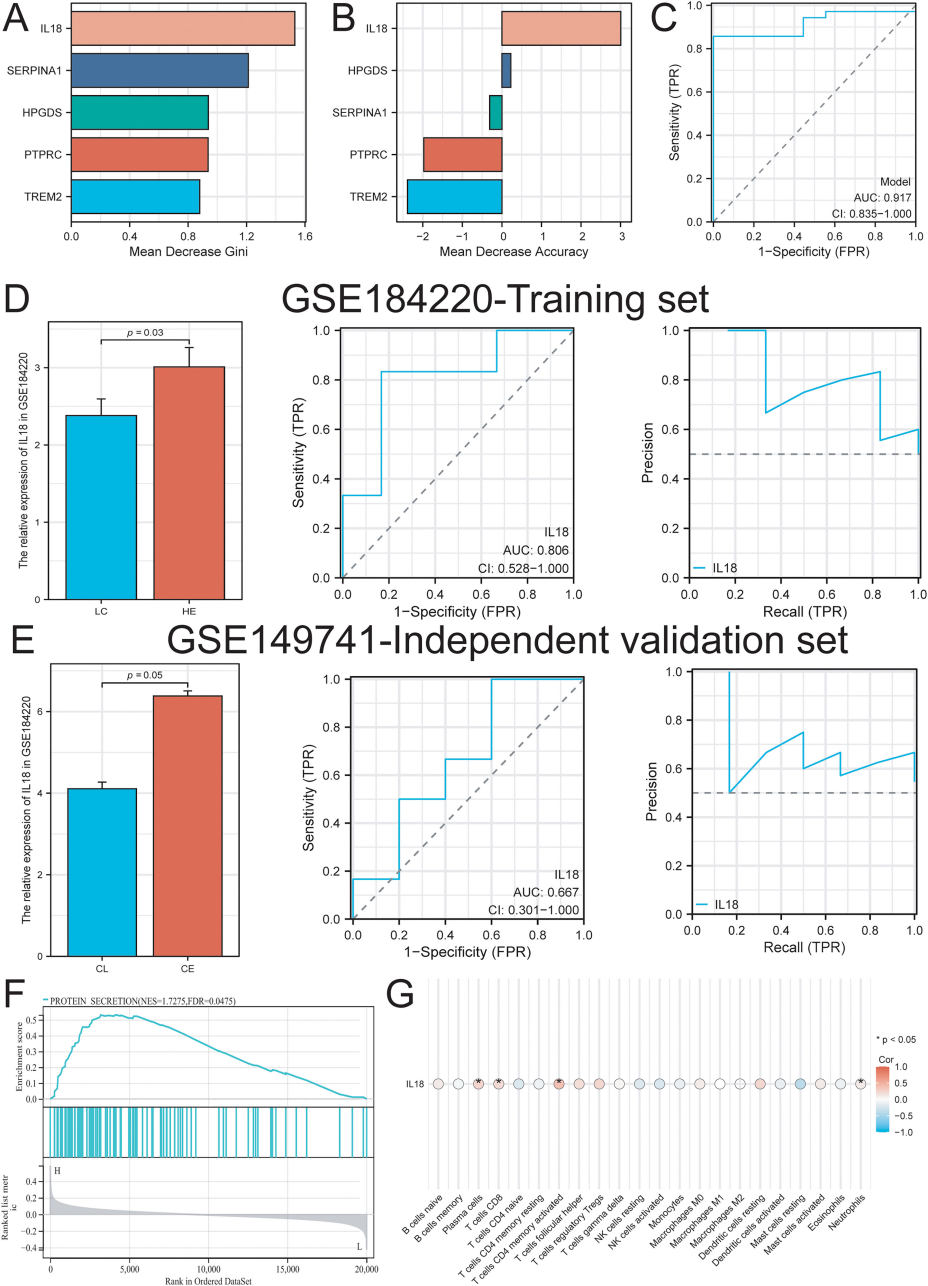
CA-associated diagnostic model construction and hub gene identification for CE patients. **(A–C**) Performance plot of the random forest model showing an AUC-index of 0.917. **(D,E)** ROC and PR curves for the model in the training set (GSE184220) and validation set (GSE149741). **(F)** Single-gene GSEA plot for IL18 in GSE184220. **(G)** Correlation heatmap between IL18 and immune cell infiltration.

Using the 5 CA-associated DEGs, we constructed a RF diagnostic model on GSE184220. The model achieved a mean AUC-index of 0.917 (Figures 3A–C). ROC analysis confirmed robust diagnostic power in the training set (AUC = 0.806) (Figure 3D), and this performance was validated in the independent GSE149741 cohort (AUC = 0.667) (Figure 3E). Single-gene GSEA in GSE184220 revealed that IL18 is primarily involved in the positive regulation of protein secretion, which potentially indicated that IL18 is a modulator for cytokine and chemokine release, ensuing leading to the chronic cerebral inflammation for CE patients (Figure 3F). Furthermore, CIBERSORT analysis revealed that high IL18 expression was positively correlated with the infiltration of plasma cells, CD8++ T cells, activated CD4+ memory T cells, and neutrophils (Figure 3G).

### 3.4 Astrocyte single-cell molecular pattern in the pathogenesis of CE patients

**FIGURE 4:**
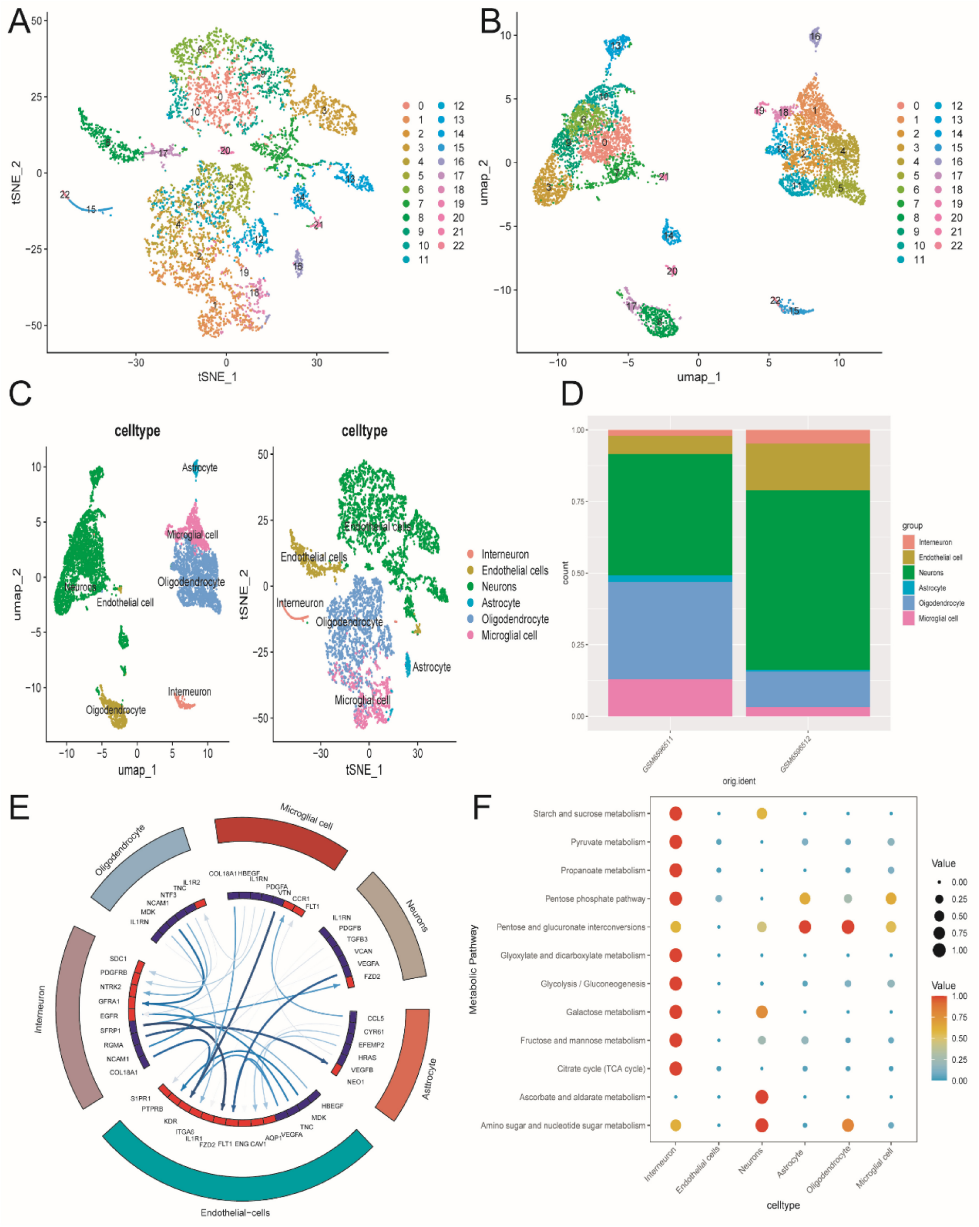
Single-cell landscape of cognitive impairment. **(A,B)** UMAP and t-SNE plots of 22 cell clusters. **(C,D)** Cell type annotation showing 6 major cell types. **(E)** CellChat network plot showing astrocyte interactions in cognitive impairment. **(F)** Heatmap of metabolic pathway activity, highlighting pentose and glucuronate interconversions in astrocytes.

Since the primary clinical manifestation of CE is cognitive impairment, we leveraged a scRNA-seq dataset of cognitive impairment (GSE163577). After quality control, 22 cell clusters were identified (Figures 4A,B and Supplementary Figure 1). These were annotated into 6 major cell types, including interneurons, endothelial cells, neurons, astrocytes, oligodendrocytes, and microglial cells (Figures 4C,D). CellChat analysis revealed strong intercellular communication involving astrocytes (Figure 4E). Metabolic profiling showed distinct heterogeneity among cell clusters, with the pentose and glucuronate interconversions pathway being specifically activated in astrocytes (Figure 4F).

### 3.5 CA-associated molecular pattern in astrocyte in the pathogenesis of CE patients

**FIGURE 5:**
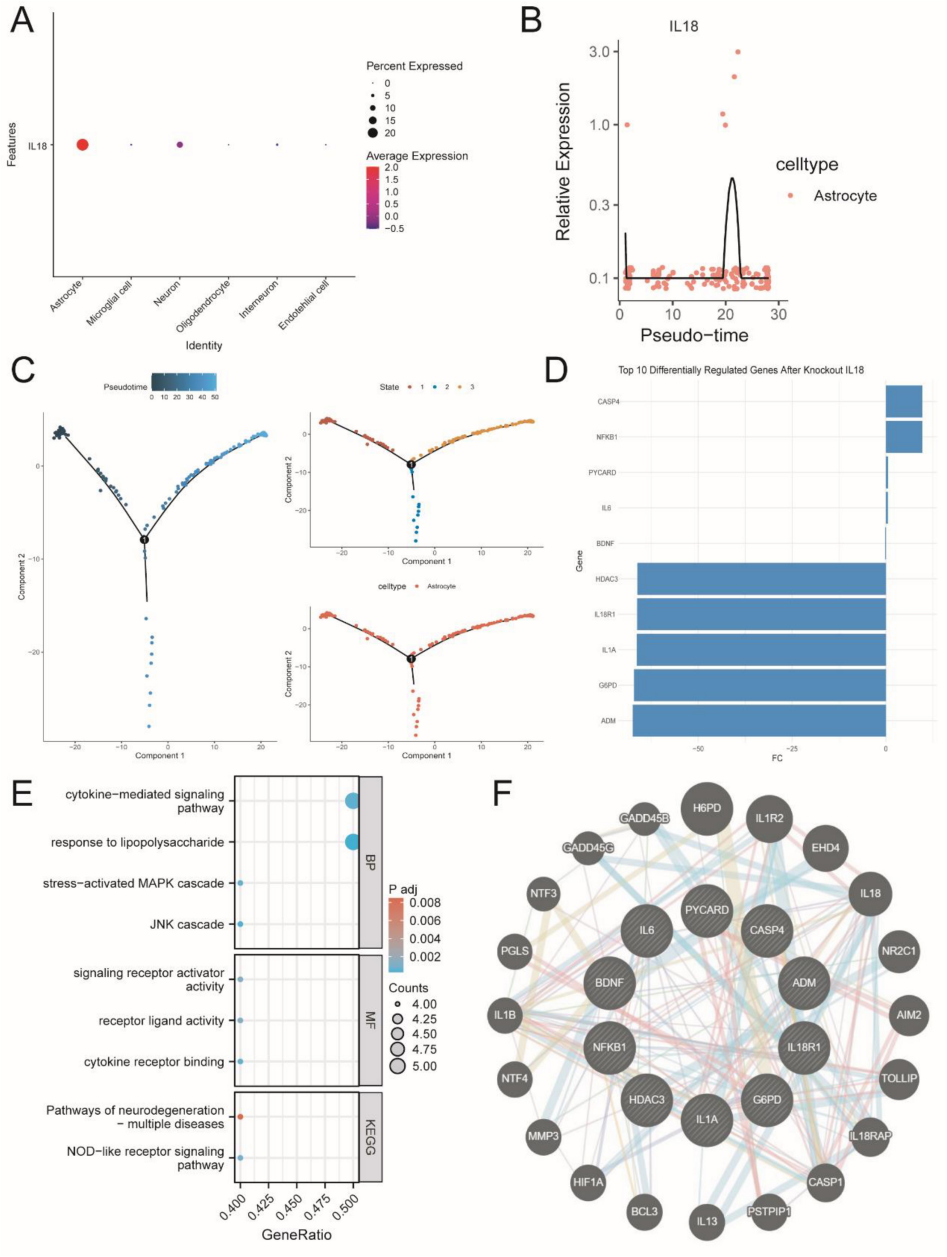
CA-associated molecular signature in astrocytes. **(A)** Feature plot showing IL18 expression is predominantly in astrocytes. **(B,C)** Pseudotime trajectory of astrocytes and corresponding IL18 expression in astrocyte differentiation. **(D–F)** Virtual knockout of IL18 in astrocyte and corresponding functional enrichment and GENEMINIA analysis.

Feature plot analysis confirmed that the expression of the hub gene IL18 was predominantly localized to the astrocyte population (Figure 5A). An *in silico* virtual knockout of IL18 specifically within astrocytes perturbed a network of genes that were subsequently enriched in pathways related to neuroinflammation and CR regulation (Figures 5D–F). Pseudotime trajectory analysis of the astrocyte cluster using Monocle2 revealed multiple differentiation branches (Figures 5B,C). Notably, the expression of IL18 was observed to gradually increase along the main differentiation trajectory, which indicated that IL18 can be considered as potential modulator in regulation of astrocyte differentiation during CE pathogenesis (Figure 5D).

### 3.6 Therapeutic agent screening for the treatment of CE targeting CA-associated molecular pattern

**FIGURE 6:**
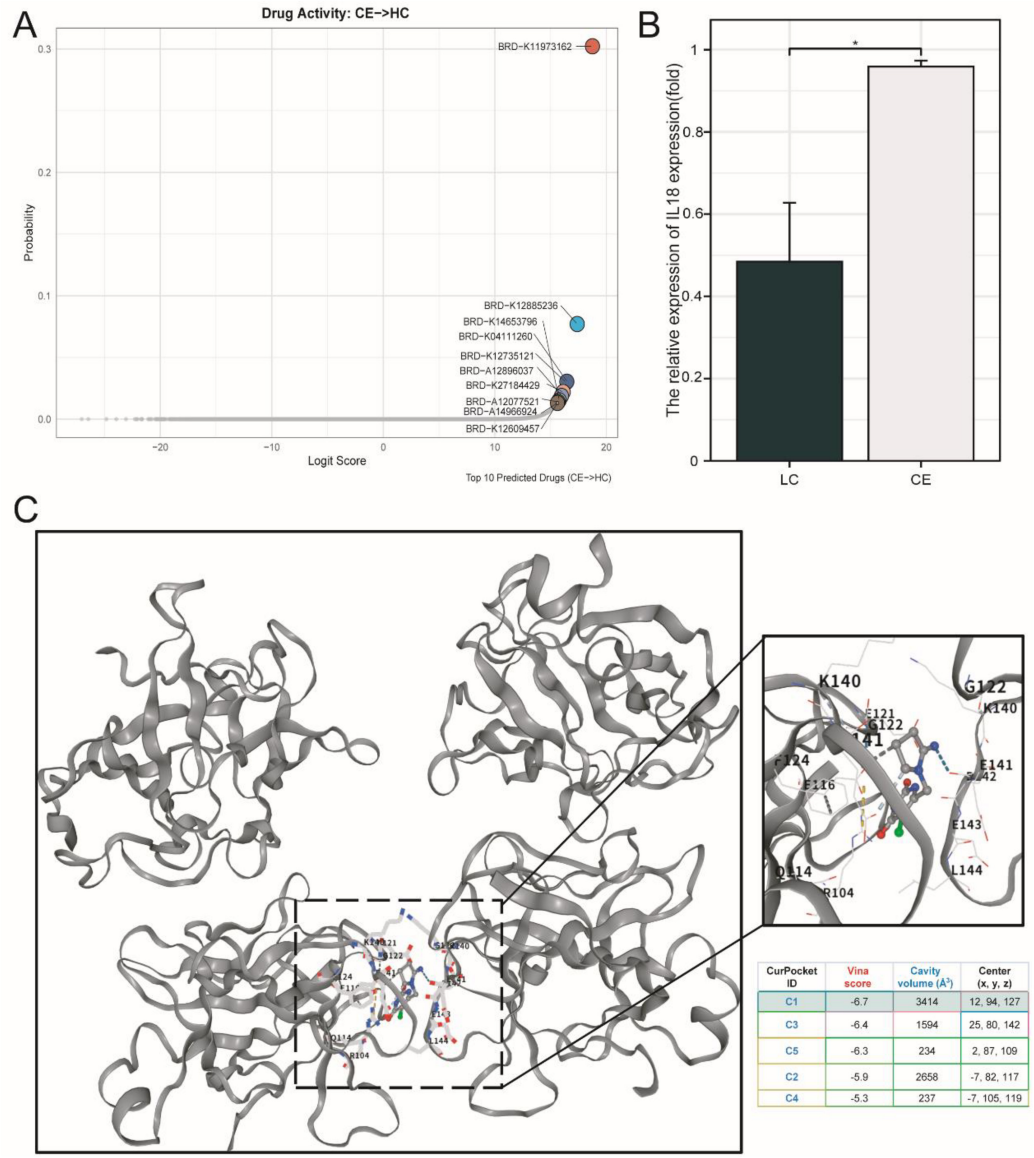
Drug screening and *in vitro* validation targeting IL18 for CE. **(A)** Bar plot from DrugReflector screening, with BRD-K11973162 as the top candidate. **(B)** *In vitro* q-RT-PCR validation (*p* < 0.05). **(C)** 3D molecular docking visualization of BRD-K11973162 with IL18. The symbol * indicates statistical significance (*p* < 0.05).

DrugReflector screening of the CE signature from GSE184220 nominated BRD-K11973162 as the top candidate compound for reversing the disease state (Figure 6A). Molecular docking predicted a strong and stable binding affinity of BRD-K11973162 to the IL18 protein (binding energy = −6.7 kcal/mol) (Figure 6C). Finally, q-RT-PCR analysis of CE astrocyte cell line compared to normal astrocyte cell line confirmed that IL18 mRNA levels were significantly upregulated in CE group compared to HC controls (*p* < 0.01) (Figure 6B), validating its potential as a circulating biomarker.

## 4 Discussion and conclusion

This study identified a novel molecular co-expression pattern connecting CA function in the pathogenesis of CE. By integrating bulk transcriptomics, single-cell resolution mapping, and AI-driven analysis, we identified IL18 as a central hub gene within the CA signature, predominantly expressed in astrocytes.

Interleukin-18 (IL18) is a pro-inflammatory cytokine belonging to the IL-1 family, primarily known for its role in inducing interferon-gamma production and promoting Th1 and Th17 immune responses (Zhang et al., 2022). Beyond its canonical function, IL18 has emerged as a key player in the central nervous system, where it can be produced by glial cells, including astrocytes and microglia, in response to injury and inflammation (Yamanishi et al., 2023). Elevated IL18 levels have been implicated in the pathogenesis of several neurodegenerative and neuroinflammatory conditions, including Alzheimer’s disease, multiple sclerosis, and traumatic brain injury (Wang et al., 2024). In the context of CE, systemic inflammation driven by gut-derived toxins is a known trigger of neurological dysfunction (Karanfilian et al., 2020). Our study provides the first evidence specifically connecting IL18 to CE pathology via the astrocyte compartment. *in silico* knockout experiments suggest that IL18 in astrocytes is not merely a marker but a functional driver, perpetuating a cycle of neuroinflammation and further disrupting the circadian clock machinery. The downregulation of IL18 along the astrocyte pseudotime trajectory in a non-CE cognitive impairment model suggests a specific, potentially chronic activation state in CE that is distinct.

In conclusion, this study presents a novel IL18-centered CA-associated molecular framework for understanding CE pathogenesis. This framework offers a potential molecular stratification and diagnostic model and identified a promising therapeutic target for halting the progression of this debilitating neurological disease in cirrhotic patients. However, this study has several limitations. To begin with, the diagnostic model and molecular subtypes of CE proposed in this study should be validated through clinical trials to assess patient clinical characteristics and improve model reliability. Furthermore, while single-cell profiling offers multi-level insights, the analysis relied on a limited cohort of only 9 cognitive impairment patients, which may not fully capture the heterogeneity of CR activity in astrocytes across the CE spectrum because of CE symptoms, including personality changes, abnormal behavior, inattention, memory impairment, and coma. Future work should incorporate higher-resolution single-cell and spatial transcriptomic data from CE patients, alongside preclinical studies that track dynamic CE alterations in astrocytes to clarify disease mechanisms. In addition, the exact downstream molecular pathways of IL18 through which CE in astrocytes compromises neuronal health remain incompletely defined in preclinical CE models. Subsequent research should focus on *in vivo* experiments, including conditional knockout or astrocyte-specific overexpression of IL18 in CE mouse models, to dissect the detailed mechanisms suggested by our *in silico* knockout analysis. At the same time, high-throughput screening that integrates CE patient clinical data to identify astrocyte-selective IL18 modulators could yield more targeted therapeutic options than currently available pan-inhibitors. Likewise, the therapeutic candidate identified through computational methods requires rigorous experimental validation in preclinical models of CE to confirm its efficacy, optimal dosing, blood–brain barrier (BBB) penetration, and ability to modulate the proposed CA-associated axis and IL18.

## Data Availability

The datasets presented in this study can be found in online repositories. The names of the repository/repositories and accession number(s) can be found in the article/Supplementary material.
