# Supplementary material for: Identification and validation of circadian rhythm and astrocyte-associated diagnostic and therapeutic model for cirrhosis encephalopathy patients via integrative bioinformatic pipelines and in vitro validation
Source: Front Neurosci. 2026 Jul 30;20:1897110. doi: 10.3389/fnins.2026.1897110 (PMC13469657; doi:10.3389/fnins.2026.1897110)
Supplement: Supplementary file 1 [file Data_Sheet_1.docx]

**Figure S1: Pre-processing of single-cell data.** (A-C) QC metrics of single-cell data. (D) Markers of Cell clusters.
